# Prevalence of Antimicrobial Resistance in Gram-Negative Bacteria Bloodstream Infections in Peru and Associated Outcomes: VIRAPERU Study

**DOI:** 10.4269/ajtmh.22-0556

**Published:** 2023-09-18

**Authors:** Fiorella Krapp, Coralith García, Noemi Hinostroza, Lizeth Astocondor, Claudia R. Rondon, Brecht Ingelbeen, Hugo A. Alpaca-Salvador, Catherine Amaro, Carla Aguado Ventura, Evelyn Barco-Yaipén, Cesar Bocangel Fernandez, Alexander Briones, Antonio Burgos, Rene Campana, Kelly Castillo, Alex Castañeda-Sabogal, Angelica Coaquira, Fátima Concha-Velasco, Edwin Cuaresma Cuadros, Omayra Chincha, Juan Carlos Diaz, Roberto Díaz Sipión, Victor Fernandez, Miguel Hueda-Zavaleta, Enrique López, María Valera-Krumdieck, Rubén Vásquez, Ana María Vidaurre Torres, Miguel Villegas-Chiroque, Favio Sarmiento Lopez, Pedro Alberto Sullón Zavaleta, Elizett Sierra Chavez, Eduardo Paricahua Peralta, Teresa Peralta Córdova, Jimena Edith Pino-Dueñas, Jan Jacobs

**Affiliations:** ^1^ Instituto de Medicina Tropical Alexander von Humboldt and School of Medicine, Universidad Peruana Cayetano Heredia, Lima, Peru;; ^2^ Department of Microbiology, Immunology and Transplantation, KU Leuven, Leuven, Belgium;; ^3^ Department of Infectious and Tropical Diseases and Dermatology, Hospital Cayetano Heredia, Lima, Peru;; ^4^ Instituto de Medicina Tropical Alexander von Humboldt, Universidad Peruana Cayetano Heredia, Lima, Peru;; ^5^ Institute of Tropical Medicine Antwerp, Antwerp, Belgium;; ^6^ Julius Center for Health Sciences and Primary Care, Utrecht University, Utrecht, the Netherlands;; ^7^ Servicio de Patología Clínica, Hospital III de Chimbote de EsSalud Ancash, Chimbote, Peru;; ^8^ Escuela de Medicina, Universidad Nacional del Santa, Chimbote, Peru;; ^9^ Departamento de Patología Clínica y Anatomía Patológica, Hospital Cayetano Heredia, Lima, Peru;; ^10^ Laboratorio Central, Hospital Regional de Ica, Ica, Peru;; ^11^ Departamento de Patología Clínica, Hospital Regional José Alfredo Mendoza Olavarría, Tumbes, Peru;; ^12^ Departamento de Medicina Interna, Hospital III Goyeneche, Arequipa, Peru;; ^13^ School of Medicine, Universidad Nacional de San Agustín, Arequipa, Peru;; ^14^ Departamento de Patología Clínica y Anatomía Patológica, Hospital Regional de Loreto Felipe Santiago Arriola Iglesias, Iquitos, Peru;; ^15^ Departamento de Patología Clínica y Anatomía Patológica, Hospital Regional de Pucallpa, Pucallpa, Peru;; ^16^ Departamento de Patología Clínica y Anatomía Patológica, Hospital III Goyeneche, Arequipa, Peru;; ^17^ Departamento de Patología Clínica y Anatomía Patológica, Hospital Belén de Trujillo, Trujillo, Peru;; ^18^ Departamento de Medicina, Hospital Base Víctor Lazarte Echegaray de EsSalud La Libertad, Trujillo, Peru;; ^19^ School of Medicine, Universidad Cesar Vallejo, Trujillo, Peru;; ^20^ Departamento de Patología Clínica y Anatomía Patológica, Hospital Santa Rosa de Puerto Maldonado, Madre de Dios, Peru;; ^21^ Departamento de Medicina, Hospital Antonio Lorena, Cusco, Peru;; ^22^ Departamento de Medicina, Universidad Nacional San Antonio Abad del Cusco, Cusco, Peru;; ^23^ Departamento de Ayuda al Diagnóstico y Tratamiento, Hospital III Daniel Alcides Carrión EsSalud Tacna, Tacna, Peru;; ^24^ Departamento de Medicina, Hospital Regional de Ica, Ica, Peru;; ^25^ School of Medicine, Universidad San Luis Gonzaga de Ica, Ica, Peru;; ^26^ Departamento de Ayuda al Diagnóstico y Tratamiento, Hospital Regional Lambayeque, Chiclayo, Peru;; ^27^ Departamento de Medicina Interna, Hospital Belén de Trujillo, Trujillo, Peru;; ^28^ Departamento de Medicina, Hospital III Daniel Alcides Carrión Essalud Tacna, Tacna, Peru;; ^29^ Facultad de Ciencias de la Salud, Universidad Privada de Tacna, Tacna, Peru;; ^30^ Departamento de Medicina, Hospital Regional de Loreto Felipe Santiago Arriola Iglesias, Iquitos, Peru;; ^31^ Departamento de Patología Clínica y Anatomía Patológica, Hospital María Auxiliadora, Lima, Peru;; ^32^ Servicio de Infectología y Medicina Tropical, Hospital María Auxiliadora, Lima, Peru;; ^33^ Departamento de Medicina, Hospital III de Chimbote EsSalud Ancash, Chimbote, Peru;; ^34^ Facultad de Ciencias de la Salud, Universidad Cesar Vallejo, Chimbote, Peru;; ^35^ Departamento Áreas Clínicas, Hospital Regional Lambayeque, Chiclayo, Peru;; ^36^ Departamento de Medicina, Hospital Regional de Pucallpa, Pucallpa, Peru;; ^37^ Departamento de Especialidades Médicas, Hospital Nacional Hipólito Unanue, Lima, Peru;; ^38^ Departamento de Patología Clínica y Anatomía Patológica, Hospital Nacional Hipólito Unanue, Lima, Peru;; ^39^ Departamento de Medicina, Hospital Santa Rosa de Puerto Maldonado, Madre de Dios, Peru;; ^40^ Departamento de Ayuda al Diagnóstico y Tratamiento, Hospital Base Víctor Lazarte Echegaray de EsSalud La Libertad, Trujillo, Peru;; ^41^ UPSS Patología Clínica, Servicio de Microbiología, Hospital Antonio Lorena Cusco, Cusco, Peru

## Abstract

Surveillance of antimicrobial resistance among gram-negative bacteria (GNB) is of critical importance, but data for Peru are not available. To fill this gap, a non-interventional hospital-based surveillance study was conducted in 15 hospitals across Peru from July 2017 to October 2019. Consecutive unique blood culture isolates of key GNB (*Escherichia coli*, *Klebsiella pneumoniae*, *Pseudomonas aeruginosa*, *Acinetobacter* spp.) recovered from hospitalized patients were collected for centralized antimicrobial susceptibility testing, along with linked epidemiological and clinical data. A total of 449 isolates were included in the analysis. Resistance to third-generation cephalosporins (3GCs) was present in 266 (59.2%) GNB isolates. Among *E. coli* (*n =* 199), 68.3% showed 3GC resistance (i.e., above the median ratio for low- and middle-income countries in 2020 for this sustainable development goal indicator). Carbapenem resistance was present in 74 (16.5%) GNB isolates, with wide variation among species (0% in *E. coli*, 11.0% in *K. pneumoniae*, 37.0% in *P. aeruginosa*, and 60.8% in *Acinetobacter* spp. isolates). Co-resistance to carbapenems and colistin was found in seven (1.6%) GNB isolates. Empiric treatment covered the causative GNB in 63.3% of 215 cases. The in-hospital case fatality ratio was 33.3% (92/276). *Pseudomonas aeruginosa* species and carbapenem resistance were associated with higher risk of in-hospital death. In conclusion, an important proportion of bloodstream infections in Peru are caused by highly resistant GNB and are associated with high in-hospital mortality.

## INTRODUCTION

Antimicrobial resistance is the third leading cause of death worldwide, responsible for at least 1.27 million deaths in 2019.^1^ Low- and middle- income countries (LMICs) are disproportionally affected by antimicrobial resistance.^2^ Consequently, antimicrobial resistance represents a serious threat to the world’s sustainable development,^3^ not only because of its burden of disease but also because of its potential to intensify global health and economic inequality.^1^^,^^2^^,^^4^

Gram-negative bacteria (GNB) are of special concern because resistance to last-resort antibiotics is emerging and spreading rapidly worldwide.^5^ In fact, four out of the six leading pathogens contributing to the burden of antimicrobial resistance in 2019 were GNB (*Escherichia coli*, *Klebsiella pneumoniae*, *Acinetobacter baumannii*, and *Pseudomonas aeruginosa*).^1^ These pathogens are also considered of critical priority for research and development of new antibiotics.^6^ Importantly, bloodstream infections caused by multidrug-resistant GNB are considered one of the most life-threatening infections, with a mortality ranging from 32–43%.^7^^,^^8^

To inform local antibiotic treatment guidelines and help close antimicrobial resistance data gaps in LMICs, we conducted a surveillance study in hospitals across Peru, with the aim of collecting representative and accurate data to determine the distribution and antimicrobial resistance profiles of four key GNB bloodstream infections. In addition, by collecting individual patient data and clinical outcomes, we aimed to provide initial data on the coverage (i.e., in vitro activity) of administered empiric antibiotic treatment and excess in-hospital mortality associated with bloodstream infections caused by specific antimicrobial resistance profiles.

## MATERIALS AND METHODS

**Figure 1.**
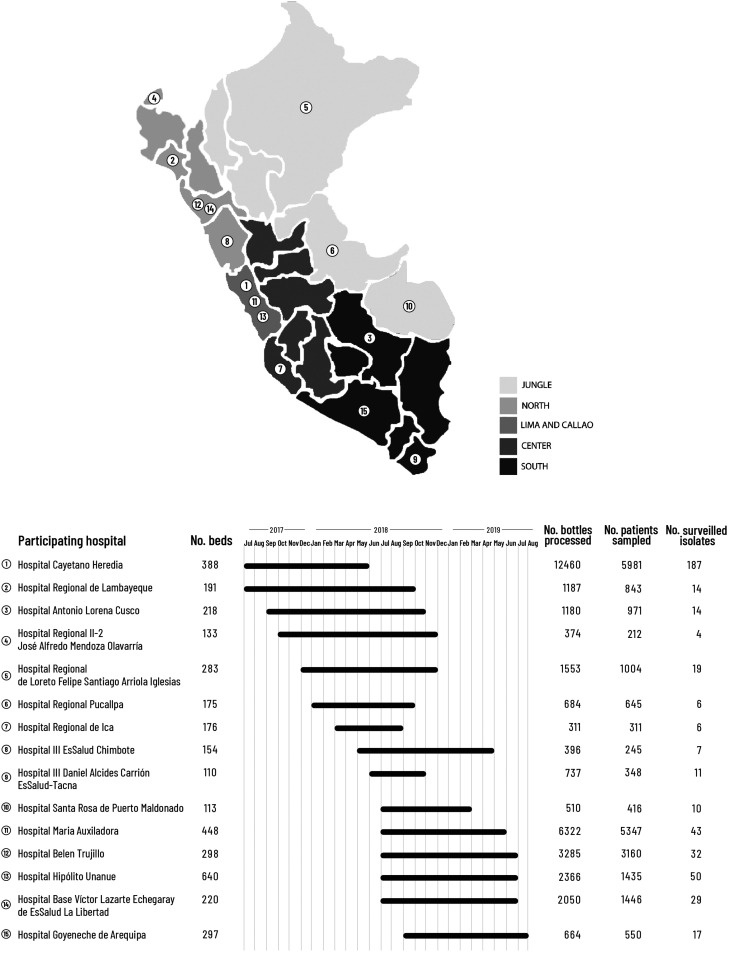
Enrolled public tertiary care hospitals as sentinel hospitals to the VIRAPERU surveillance study and their location within the 24 regions and 5 macro regions of Peru. The number of hospital beds, time period of participation, number of blood cultures processed, and number of surveilled gram-negative bacteria isolates are presented for each hospital.

From July 2017 to October 2019, we conducted a multicenter prospective hospital-based surveillance study of *E. coli*, *K. pneumoniae, Acinetobacter* spp., and *P. aeruginosa* (key GNB) recovered from routine blood cultures submitted from hospitalized adults and children. Fifteen tertiary care public hospitals from 12 out of 24 regions (and from all five macro regions) of Peru were enrolled as sentinel hospitals in a staggered fashion and followed up for 6 to 12 months (Figure 1). These hospitals had adult and pediatric wards, intensive care units (ICUs), and a clinical microbiology laboratory routinely performing blood cultures, most of them with automated blood culture systems. Requests for blood cultures were made by treating physicians based on clinical judgment. At each hospital, a laboratory and a clinical coinvestigator were trained on study procedures that included standardized collection, storage, and transport of isolates and collection of clinical and epidemiological data, respectively.

### Aggregated data collection.

On a monthly basis, the laboratories’ coinvestigators reported the number of patients with a blood culture submitted; processed blood culture bottles; bottles with any growth; coagulase-negative staphylococci growth (used as a proxy of contamination); and growth of each of the four key GNB.

### Isolates collection.

Participating laboratories stored blood isolates belonging to the four key GNB. Isolates were stored in Tryptic Soy Agar vials at room temperature. First (i.e., nonduplicate) isolates were transported monthly to the reference laboratory at Instituto de Medicina Tropical Alexander von Humboldt in Lima, along with their original identification and antimicrobial susceptibility testing results. Hospitals were instructed to submit only the first isolate per patient.

### Individual patient data collection.

Once a key GNB was identified, the clinical coinvestigator was informed by the laboratory to localize the medical chart of the source patient and to collect the following data: age, sex, comorbidities, date of hospital admission, date of ICU admission and date of blood culture sampling, and in-hospital outcome (discharge versus death). Age group was assigned as “neonate” if patients were 1 month old or younger, “pediatric” if older than 1 month and younger than 18 years, and “adult” if 18 years old or older.

In addition, for adult patients, comorbidities and data on disease severity (Pitt bacteremia score^9^ and quick Sequential Organ Failure Assessment score [qSOFA]^10^) were registered. Except for neonates, the origin of bloodstream infection was registered following the Global Antimicrobial Resistance and Use Surveillance System (GLASS)/WHO classification^11^ as 1) community origin if blood culture was sampled ≤ 2 days after hospital admission or 2) hospital origin if blood culture was sampled > 2 days after hospital admittance.

Empiric treatment was defined as any antibiotic received (at least one dose) within the first 2 calendar days after blood culture sampling, with day of sampling defined as day 1. Empiric antibiotics with gram-negative coverage were later categorized as ACCESS, WATCH, or RESERVE based on the WHO AWaRe Classification (tool developed for antibiotic surveillance and stewardship that classifies more than 100 antibiotics as first-line [ACCESS], second-line [WATCH], or last resort [RESERVE] for treatment of common infectious syndromes, taking into consideration their resistance potential).^12^ If a patient received more than one antibiotic from different categories, the highest category was considered. Activity of empiric treatment was assessed based on the reference identification and antimicrobial susceptibility testing results of the recovered isolate. If the isolate was resistant to all antibiotics received, the empiric treatment was classified as “inactive.” If it was intermediate or susceptible to at least one antibiotic received, it was classified as “active.” In the rare cases where no antimicrobial susceptibility result was available for a specific administered antibiotic, this was classified as inactive if a tested antibiotic of the same antibiotic class was resistant.

### Reference identification and antimicrobial susceptibility testing.

Reference identification and antimicrobial susceptibility testing were performed at the reference laboratory. Identification was performed by conventional biochemical testing^13^; for isolates identified as *Acinetobacter* spp., polymerase chain reaction was performed to detect the *blaOXA-51* gene for identification of *A. baumannii* species.^14^

Each retrieved isolate confirmed as belonging to one of the four key GNB species underwent testing with a standardized panel of 12 to 16 antibiotics, according to the identified species (Supplemental Table 1). Antibiotics were included in these panels if 1) they were listed as priority pathogen-antibiotic combinations in GLASS^11^ or 2) they belonged to the group A or B antibiotics of the Clinical and Laboratory Standards Institute (CLSI) M100 S27 guideline^15^ and were of local clinical relevance. The disk diffusion method was conducted following the M100 CLSI guideline valid at the time of testing, and results were interpreted using 2021 CLSI breakpoints,^16^ except for fosfomycin against *P. aeruginosa* and tigecycline, for which the European Committee on Antimicrobial Susceptibility Testing^17^ and the U.S. Food and Drug Administration^18^ breakpoints were used, respectively. Colistin susceptibility testing was performed only for carbapenem-resistant isolates, using both colistin agar spot^19^ and disk elution methods and applying 2021 CLSI breakpoints.^16^ Extended-spectrum beta-lactamase (ESBL) production was assessed only for *E. coli* and *K. pneumoniae* isolates resistant to third-generation cephalosporins (3GCs) using the double disk method.^16^ *Escherichia coli* American Type Culture Collection (ATCC) 25922, *P. aeruginosa* ATCC 27853, and *Proteus mirabilis* ATCC 12453 strains were used for quality control. All antibiotic susceptibility tests were performed once per isolate, except in the case of out-of-range quality control results, in which case a second test was performed using the entire panel of antibiotics. Resistance to an antibiotic class was defined as resistance to at least one tested antibiotic of the corresponding class (i.e., ceftriaxone or ceftazidime for 3GC; ciprofloxacin for fluoroquinolones; amikacin or gentamicin for aminoglycosides; meropenem, imipenem, or ertapenem for carbapenems). Multidrug resistance (MDR) was defined as acquired nonsusceptibility (intermediate or resistant) to at least one antibiotic in three or more antibiotic classes.^20^ Difficult-to-treat resistance (DTR) was defined as combined nonsusceptibility (intermediate or resistant) to all tested beta-lactam antibiotics (including carbapenems) and ciprofloxacin.^8^

### Data analysis.

We determined the frequency of resistance (i.e., number of resistant isolates divided by number of all tested isolates) to each antibiotic and to specific antibiotic classes (3GCs, carbapenems, fluoroquinolones, and aminoglycosides) in addition to the prevalence of DTR. Possible clustering was evaluated by identifying isolates that were recovered in the same ward within a 14-day span and had similar resistance profiles.

Demographic and clinical characteristics of the source patients, including activity of empiric treatment and hospitalization outcomes, were described, determining the frequency for categorical variables and the median with interquartile range (IQR) for continuous variables. A stratified analysis of the frequencies of resistance, activity of empiric treatment, and hospitalization outcomes (in-hospital case fatality ratio and length of hospital stay) was also conducted based on the origin of bloodstream infection (community versus hospital), age group (adult, pediatric, and neonate), and hospital ward (ICU versus non-ICU). We also compared frequencies of resistance between isolates recovered from Lima, where resources are usually centralized, versus the other macro regions.

Bivariate regression analyses were conducted to explore the association of species, origin of infection, and specific antimicrobial resistance profiles (3GC and carbapenem resistance, DTR) with 1) inactivity of empiric treatment and 2) in-hospital mortality. For this, a generalized linear model considering the negative binomial discrete distribution and the logarithmic function was used to estimate crude RRs with 95% CIs. In addition, a competing-risks analysis, using discharge as a competing event of in-hospital death, was used to compare the cumulative incidence functions of in-hospital fatality after blood culture collection between patients with 3GC-resistant and those with nonresistant *E. coli* bloodstream infections, between carbapenem-resistant and nonresistant GNB bloodstream infections, and between DTR and non-DTR GNB bloodstream infections. A value of *P* < 0.05 was used to determine statistical significance.

## RESULTS

**Figure 2.**
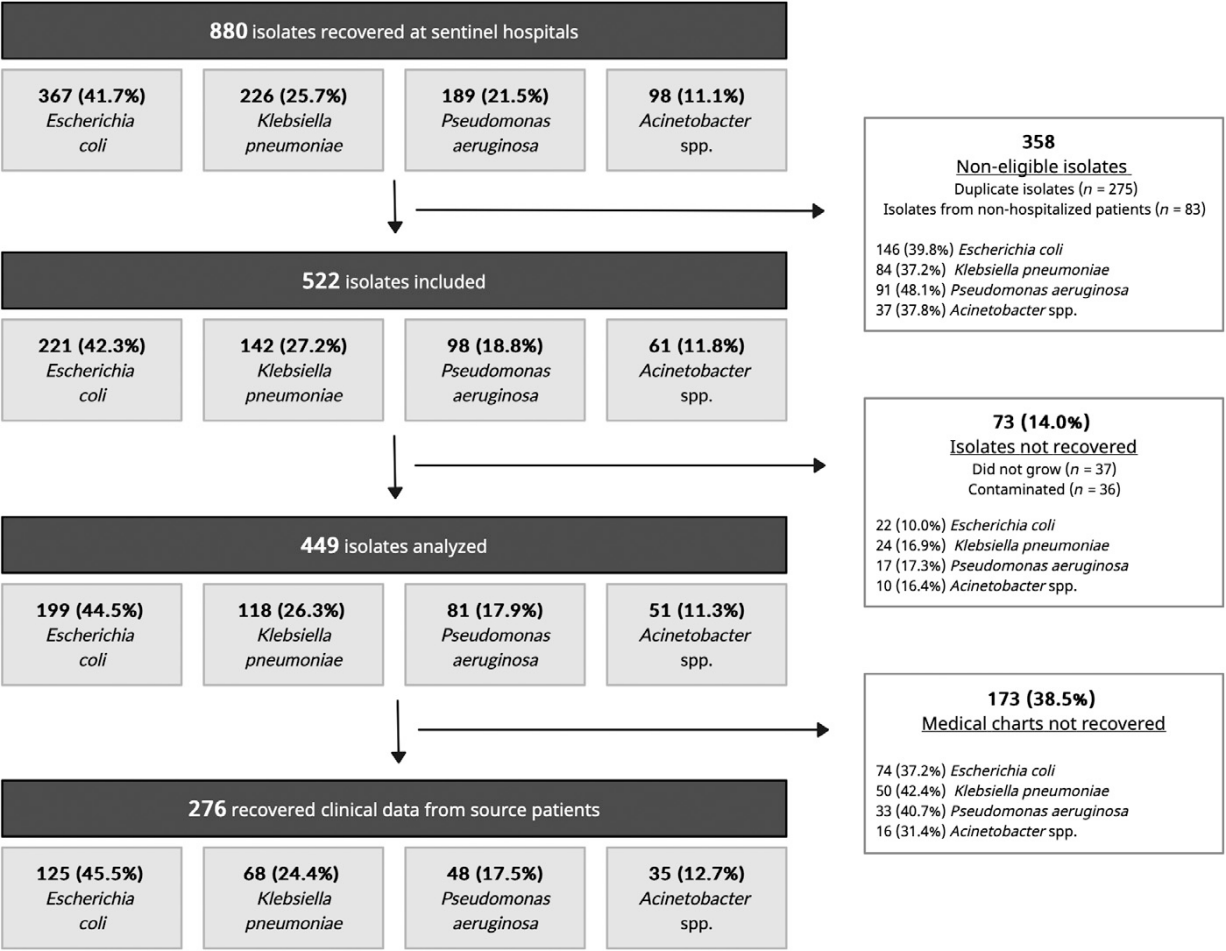
Number of gram-negative bacteria blood isolates recovered from hospitalized patients at sentinel hospitals that met the inclusion criteria and that were analyzed in the present surveillance study.

During the study period, 34,079 blood culture bottles from 22,914 patients were processed in the laboratories of the sentinel hospitals. A total of 4,732 (13.9%) bottles showed growth, with 2,630 (7.7%) coagulase-negative staphylococci (used as a proxy of blood culture contamination) and 2,102 (6.2%) pathogens. Overall, the four key GNB accounted for 41.9% (*n =* 880) of pathogens recovered. Other frequently recovered pathogens were *Staphylococcus aureus* in 19.6% (*n =* 411) and *Candida* spp. in 10.1% (*n =* 212). A total of 522 GNB isolates met the inclusion criteria of being the first isolate per hospitalized patient diagnosed with a bloodstream infection. From the 522 included GNB isolates, 37 (7.1%) did not grow upon retrieval from storage and 36 (6.9%) were contaminated; this occurred randomly across hospitals and species. Therefore, a total of 449 isolates underwent antimicrobial susceptibility testing to the prespecified panel of antibiotics. These included 199 *E. coli*, 118 *K. pneumoniae*, 81 *P. aeruginosa*, and 51 *Acinetobacter* spp. isolates, from which 38 (74.5%) belonged to the *A. baumannii* species (Figure 2).

### Frequency of antimicrobial resistance in GNB bloodstream infections.

All 449 isolates were tested to their respective antibiotic panels, resulting in a total of 6,281 antibiotic susceptibility tests performed (Supplemental Table 2). Overall, resistance to at least one antibiotic was found in 365 (81.3%) isolates and MDR was found in 310 (69.0%) (Table 1). Resistance to 3GCs, carbapenems, and fluoroquinolones was found in 266 (59.2%), 74 (16.5%), and 285 (63.5%) GNB, respectively, with wide variations between species (Table 1). For instance, carbapenem resistance was not found among *E. coli*, compared with a prevalence of 11.0% among *K. pneumoniae*, 37.0% among *P. aeruginosa*, and 60.8% among *Acinetobacter* spp. The overall prevalence of DTR was 10.2%, being highest for *Acinetobacter* spp. (49.0%) and absent among *E. coli* (Table 1). Carbapenem resistance and DTR were even higher among *A. baumannii* isolates, reaching 71.1% (27/38) and 60.5% (23/38). Co-resistance to carbapenems and colistin was found in 7 (1.6%) and 22 (4.9%) isolates when tested by the agar spot or disk elution method, respectively.

**Table 1:** Frequency of resistance to different antibiotics among the four key recovered gram-negative bacteria causing bloodstream infection

Antibiotic	*Escherichia coli* (*n =* 199)		*Klebsiella pneumoniae* (*n =* 118)		*Pseudomonas aeruginosa* (*n =* 81)		*Acinetobacter* spp. (*n =* 51)		Total (*N =* 449)	
	*n*	%	*n*	%	*n*	%	*n*	%	*n*	%
Resistance profile										
Pan-susceptible	10	5.0	17	14.4	21	25.9	13	25.5	61	13.6
Antibiotic resistance*	179	90.0	94	79.7	56	69.1	36	70.6	365	81.3
Multidrug resistance†	155	77.9	88	74.6	33	40.7	34	66.7	310	69.0
Difficult-to-treat resistance‡	0	0.0	11	9.3	10	12.4	25	49.0	46	10.2
Resistance to 3GC	136	68.3	81	68.6	16	19.8	33	64.7	266	59.2
ESBL production§	129	94.9	66	81.5	–	–	–	–	195	89.9
Co-resistance										
3GC/4GC + quinolones	123	61.8	74	62.7	17	21.0	32	62.8	246	54.8
Carbapenems	0	0.0	13	11.0	30	37.0	31	60.8	74	16.5
Carbapenem + aminoglycoside	–	–	10	8.5	21	25.9	28	54.9	59	13.1
Carbapenem + colistin (agar spot)	–	–	2	1.7	2	2.5	3	5.9	7	1.6
Carbapenem + colistin (disk elution)	–	–	2	1.7	5	6.2	15	29.4	22	4.9
Specific antibiotic resistance										
ACCESS category										
Ampicillin-sulbactam	–	–	–	–	–	–	26	51.0	–	–
Cefazolin	148	74.4	84	71.2	–	–	–	–	–	–
Gentamicin	74	37.2	58	49.2	22	27.2	26	51.0	–	–
Amikacin	13	6.5	5	4.2	17	21.0	29	56.9	–	–
Trimethoprim-sulfamethoxazole	131	65.8	78	66.1	–	–	31	60.8	–	–
WATCH category										
Ceftriaxone	136	68.3	81	68.6	–	–	–	–	–	–
Ceftazidime	86	43.2	69	58.5	16	19.8	33	64.7	–	–
Cefepime	106	53.3	64	54.2	17	21.0	–	–	–	–
Piperacillin-tazobactam	6	3.0	21	17.8	10	12.4	–	–	–	–
Ertapenem	0	0.0	13	11.0	–		–	–	–	–
Imipenem	0	0.0	8	6.8	28	34.6	29	56.9	–	–
Meropenem	0	0.0	11	9.3	23	28.4	30	58.8	–	–
Ciprofloxacin	150	75.4	80	67.8	22	27.2	33	64.7	–	–
RESERVE category										
Aztreonam	120	60.3	77	65.3	18	22.2	–	–	–	–
Fosfomycin‖	24	12.1	6	5.1	34	42.0	–	–	–	–
Tigecycline¶	0	0.0	3	2.5	–	–	4	7.8	–	–
Minocycline	–	–	–	–	–	–	4	7.8	–	–

3GC = third-generation cephalosporin; 4GC = fourth-generation cephalosporin; ESBL = extended-spectrum beta-lactamase. Categories refer to the WHO ACCESS, WATCH, and RESERVE classifications of antibiotics for evaluation and monitoring of use (AWaRE Classification).

* Defined as resistance to at least one tested antibiotic.

† Defined as nonsusceptibility (intermediate or resistant) to at least one antibiotic in three or more antibiotic classes.

‡ Difficult-to-treat resistance was defined as nonsusceptibility (intermediate and resistant) to all tested beta-lactam antibiotics, including carbapenems, and to ciprofloxacin.

§ Only tested on *E. coli* and *K. pneumoniae* isolates resistant to 3GCs.

‖ Interpretation using Clinical and Laboratory Standards Institute breakpoints for *E. coli* and *K. pneumoniae* and European Committee on Antimicrobial Susceptibility Testing epidemiologic cutoff value for *P. aeruginosa.*

¶ Interpretation according to cutoff values proposed by the U.S. Food and Drug Administration.

Higher frequencies of resistance to fluoroquinolones and aminoglycosides were found in Lima compared with other regions (78.1% and 75.0% versus 42.1% and 36.8%, *P* = 0.015 and 0.009, respectively). In addition, carbapenem resistance in *K. pneumoniae* was found only in Lima (14.7%) and in the North macro region (8.7%), but not in the other regions (Supplemental Table 3).

### Epidemiological and clinical characteristics of GNB bloodstream infections.

From the 449 included isolates, clinical data of 276 (61.5%) patients were available. Among those, 143 (51.8%) were male and 224 (81.2%) were adults. The proportion of patients with missing clinical data was similar between the different age, sex and pathogen categories (Supplemental Table 4). A low percentage (less than 20%) of isolates were recovered from children and neonates for all pathogens, except for *K. pneumoniae* bloodstream infections, for which 25 (36.8%) of the patients were children or neonates.

Comorbidity was documented in 127 (56.7%) adult patients, with diabetes being the most frequent (23.2%). Infection severity varied widely, with a median Pitt bacteremia score of 2 (range: 0 to 13) and 78 (37.1%) patients having a critical (≥ 4) score. Among patients with *P. aeruginosa* and *Acinetobacter* spp. bloodstream infections, percentages of patients with critical illness were highest (51.3% and 46.4%, respectively) (Table 2).

**Table 2:** Demographic and clinical characteristics of hospitalized patients with bloodstream infection caused by the key gram-negative bacteria surveilled

Characteristics	*N*	Total (*N =* 276)		*Escherichia coli* (*n =* 125)		*Klebsiella pneumoniae* (*n =* 68)		*Pseudomonas aeruginosa* (*n =* 48)		*Acinetobacter* spp. (*n =* 35)	
		*n*	%	*n*	%	*n*	%	*n*	%	*n*	%
Age, years, median (IQR)	276	57.5	29–72	65	46–79	35.5	0–62	53	28–71.5	54	25–71
Neonatal		32	11.6	9	7.2	17	25.0	3	6.3	3	8.6
Pediatric		20	7.2	5	4.0	8	11.8	5	10.4	2	5.7
Adult		224	81.2	111	88.8	43	63.2	40	83.3	30	85.7
Sex											
Female	276	133	48.2	67	53.6	28	41.2	24	50.0	14	40.0
Male		143	51.8	58	46.4	40	58.8	24	50.0	21	60.0
Comorbidity (at least one)*	224	127	56.7	66	59.5	26	60.5	23	57.5	12	40.0
Comorbidity*											
Diabetes	224	52	23.2	29	26.1	9	20.9	10	25.0	4	13.3
Chronic renal failure		32	14.3	13	11.7	10	23.3	8	20.0	1	3.3
Cardiac disease		27	12.1	12	10.8	5	11.6	8	20.0	2	6.7
Pulmonary disease		25	11.2	12	10.8	6	14.0	5	12.5	2	6.7
Hepatic disease		11	4.9	8	7.2	2	4.7	1	2.5	0	0.0
Immunosupression		18	8.0	10	9.0	2	4.7	5	12.5	1	3.3
HIV infection		11	4.9	1	0.9	3	7.0	2	5.0	5	16.7
Origin of infection†											
Community	244	79	32.4	55	47.4	11	21.6	8	17.8	5	15.6
Hospital		165	67.6	61	52.6	40	78.4	37	82.2	27	84.4
Pitt score, median (IQR)*	210‡	2	1–5	2	1–4	2	0–4	4	0–8	3	0.5–6.5
Critically ill (score ≥ 4)	210‡	78	37.1	33	32.0	12	30.0	20	51.3	13	46.4
qSOFA score, median (IQR)*	205‡	1	1–2	1	0–2	1	1–2	1	1–2	1	1–2
Ward at time of diagnosis											
ICU	261‡	68	26.1	20	17.2	21	32.8	15	31.3	12	36.4
Non-ICU		193	73.9	96	82.8	43	67.2	33	68.7	21	63.6
Empiric antibiotic treatment within 2 days after blood culture sampling											
None	276	61	22.1	28	22.4	15	22.1	12	25.0	6	17.1
Antibiotic with any coverage		215	77.9	97	77.6	53	79.9	36	75.0	29	82.9
Antibiotic with GNB coverage		205	74.3	95	76.0	49	72.1	32	66.7	29	82.9
AWaRE category of antibiotic with GNB coverage											
ACCESS	205	12	5.9	4	4.2	3	6.1	2	6.3	3	10.4
WATCH		187	91.2	90	94.7	46	93.9	28	87.4	23	79.3
RESERVE		6	2.9	1	1.1	0	0.0	2	6.3	3	10.3
Empiric antibiotic treatment active	215	136	63.3	72	74.2	36	67.9	18	50.0	10	34.5
LOS after BSI, days, median (IQR)§	181‡	14	9–25	13	8–22	17.5	11–28.5	16	9–39	15	7–36
In-hospital case fatality ratio	276	92	33.3	32	25.6	20	29.4	25	52.1	15	42.9
Time to death after BSI, days, median (IQR)‖	92	3.5	1–15.5	3	1.5–11.5	4	1–11.5	2	0–12	13	2–16

AWaRE = WHO access, watch, reserve, classification of antibiotics for evaluation and monitoring of use; BSI = bloodstream infection; GLASS = Global Antimicrobial Resistance and Use Surveillance System; GNB = gram-negative bacteria; ICU = intensive care unit; IQR = interquartile range; LOS = length of hospital stay; qSOFA = quick Sequential Organ Failure Assessment. Data presented correspond to 276 (61.5%) patients with available information out of the total 449 source patients.

* Evaluated only for adult patients.

† Evaluated only for pediatric and adult patients using the GLASS/WHO classification.^11^ Neonates were not considered.

‡ *N* different from 276 due to missing values.

§ Evaluated only in surviving patients, as the number of days that the patient remained hospitalized after sampling of the first positive blood culture.

‖ Evaluated only in patients who died, as the number of days from the sampling of the first positive blood culture to the date of death.

Neonates excluded, two-thirds (165/244, 67.6%) of GNB bloodstream infections were categorized as Hospital and one-third (79/244, 32.4%) as Community. *Escherichia coli* bloodstream infections had a community origin in 55 (47.4%) of the cases, whereas *K. pneumoniae* in only 11 (21.6%). *Pseudomonas aeruginosa* and *Acinetobacter* spp. bloodstream infections predominantly had (82.2% and 84.4%) a hospital origin (Table 2).

Among *E. coli* and *K. pneumoniae* isolates, resistance to 3GCs, fluoroquinolones, and aminoglycosides was lower among Community isolates than among Hospital isolates, and no carbapenem resistance was found among Community isolates. Nevertheless, a high level of resistance to 3GCs was still present among Community isolates, with 62.5% among *E. coli* and 28.6% among *K. pneumoniae* isolates (Table 3).

**Table 3:** Frequency of resistance to different antibiotic classes and DTR among the four key gram-negative bacteria blood isolates surveilled, stratified by origin of infection, type of hospital ward, and age group

Resistance profile†	Origin of infection*				Hospital ward				Age group					
	Community (*n =* 83)		Hospital (*n =* 179)		Non-ICU (*n =* 333)		ICU (*n =* 92)		Neonatal (*n =* 51)		Pediatric (*n =* 28)		Adult (*n =* 363)	
	*n*	%	*n*	%	*n*	%	*n*	%	*n*	%	*n*	%	*n*	%
*Escherichia coli*														
3GC	35/56	62.5	52/65	80.0	107/161	66.5	19/25	76.0	7/13	53.9	4/10	40.0	122/173	70.5
Carbapenems	0/56	0.0	0/65	0.0	0/161	0.0	0/25	0.0	0/13	0.0	0/10	0.0	0/173	0.0
Fluoroquinolones	43/56	76.8	54/65	83.1	120/161	74.5	21/25	84.0	8/13	61.5	3/10	30.0	137/173	79.2
Aminoglycosides	26/56	46.4	34/65	52.3	63/161	39.1	10/25	40.0	4/13	30.8	6/10	60.0	69/173	39.9
Difficult-to-treat resistance	0/56	0.0	0/65	0.0	0/161	0.0	0/25	0.0	0/13	0.0	0/10	0.0	0/173	0.0
*Klebsiella pneumoniae*														
3GC	4/14	28.6	34/44	77.3	56/81	69.1	21/31	67.7	18/26	69.2	6/10	60.0	56/81	69.1
Carbapenems	0/14	0.0	7/44	15.9	7/81	8.6	6/31	19.4	2/26	7.7	1/10	10.0	10/81	12.4
Fluoroquinolones	3/14	21.4	35/44	79.6	53/81	65.4	22/31	71.0	17/26	65.4	6/10	60.0	56/81	69.1
Aminoglycosides	3/14	21.4	24/44	54.6	36/81	44.4	19/31	61.3	16/26	61.5	5/10	50.0	37/81	45.7
Difficult-to-treat resistance	0/14	0.0	5/44	11.4	7/81	8.6	4/31	12.9	2/26	7.7	1/10	10.0	8/81	9.9
*Pseudomonas aeruginosa*														
3GC	1/8	12.5	15/42	35.7	9/58	15.5	7/20	35.0	0/4	0.0	1/6	16.7	15/68	22.1
Carbapenems	3/8	37.5	21/42	50.0	17/58	29.3	11/20	55.0	1/4	25.0	3/6	50.0	24/68	35.3
Fluoroquinolones	1/8	12.5	16/42	38.1	12/58	20.7	8/20	40.0	1/4	25.0	1/6	16.7	18/68	26.5
Aminoglycosides	2/8	25.0	16/42	38.1	13/58	22.4	7/20	35.0	0/4	0.0	1/6	16.7	19/68	27.9
Difficult-to-treat resistance	1/8	12.5	8/42	19.1	7/58	12.1	3/20	15.0	0/4	0.0	1/6	16.7	9/68	13.2
*Acinetobacter* spp.														
3GC	3/5	60.0	22/28	78.6	19/33	57.6	12/16	75.0	1/8	12.5	1/2	50.0	31/41	75.6
Carbapenems	3/5	60.0	20/28	71.4	17/33	51.5	12/16	75.0	1/8	12.5	1/2	50.0	29/41	70.7
Fluoroquinolones	2/5	40.0	23/28	82.1	19/33	57.6	12/16	75.0	1/8	12.5	0/2	0.0	32/41	78.1
Aminoglycosides	3/5	60.0	21/28	75.0	18/33	54.6	11/16	68.8	0/8	0.0	0/2	0.0	31/41	75.6
Difficult-to-treat resistance	2/5	40.0	15/28	53.6	13/33	39.4	10/16	62.5	1/8	12.5	0/2	0.0	24/41	58.5

3GC = third-generation cephalosporins; DTR = difficult-to-treat resistance; GLASS = Global Antimicrobial Resistance and Use Surveillance System; ICU = intensive care unit.

* Evaluated only in pediatric and adult patients, using the GLASS/WHO classification.^11^ Neonates were not considered.

† Resistance to third-generation cephalosporins was defined as resistance to ceftriaxone or ceftazidime for *E. coli* and *K. pneumoniae* and as resistance to ceftazidime for *P. aeruginosa* and *Acinetobacter* spp. Resistance to fluoroquinolones was defined as resistance to ciprofloxacin. Resistance to aminoglycosides was defined as resistance to amikacin or gentamicin.

Higher frequencies of resistance were observed among isolates recovered from ICU patients (Table 3). *Klebsiella pneumoniae* isolates from neonates presented high frequencies of resistance, including 69.2% with resistance to 3GCs and 7.7% with resistance to carbapenems and DTR. The small number of isolates of other GNB recovered from neonatal and pediatric patients (< 10 isolates per group) limited further comparisons (Table 3). Eight possible clusters were identified (including a total of 16 isolates), with only a single one involving neonates (two *Acinetobacter* spp. isolates) and none involving ICU patients (Supplemental Table 5).

### Empiric treatment activity.

From the 276 patients with clinical data available, 215 (77.9%) received at least one antibiotic as empiric treatment, and 205 (74.3%) received at least one antibiotic with gram-negative spectrum. Empiric gram-negative treatment contained RESERVE antibiotics in 2.9% of the patients and WATCH antibiotics in 91.2%, with only 5.9% of patients receiving ACCESS antibiotics (Table 2).

Overall, empiric treatment was active in 136 (63.3%) of the cases. Empiric treatment with gram-negative spectrum tended to be more frequently active against *E. coli* and *K. pneumoniae* bloodstream infections (75.8% and 73.5%, respectively) than against *P. aeruginosa* (56.2%) and *Acinetobacter* spp. (34.5%) bloodstream infections (Table 4), although only *Acinetobacter* spp. bloodstream infections were associated with a higher risk of inactive empiric treatment (RR: 2.71, 95% CI: 1.30–5.65; *P* = 0.008). The risk of receiving inactive GNB empiric treatment was more than three times higher if bloodstream infection presented 3GC resistance (RR: 3.61, 95% CI: 1.69–7.69; *P* = 0.001), carbapenem resistance (RR: 4.03, 95% CI: 2.23–7.26; *P* < 0.001), or DTR (RR: 3.89, 95% CI: 1.99–7.63; *P* < 0.001) (Table 4).

**Table 4:** Inactivity of empiric treatment and case fatality ratios according to different antimicrobial resistance profiles

Characteristics	Inactivity of GNB empiric treatment							Total case fatality ratio						
	Inactive (*n =* 69)		Active (*n =* 136)		Bivariate regression			Survived (*n =* 184)		Died (*n =* 92)		Bivariate regression		
	*n*	%	*n*	%	RR	95% CI	*P*	*n*	%	*n*	%	RR	95% CI	*P*
Age group														
Neonatal	5	20.8	19	79.2	1.00	–	–	24	75.0	8	25.0	1.00	–	–
Pediatric	5	31.3	11	68.7	1.50	0.37–6.03	0.568	16	80.0	4	20.0	0.80	0.21–3.01	0.741
Adult	59	35.8	106	64.2	1.72	0.63–4.70	0.294	144	64.3	80	35.7	1.43	0.63 –3.23	0.391
Sex														
Female	34	34.0	66	66.0	1.00	–	–	87	65.4	46	34.6	1.00	–	–
Male	35	33.3	70	66.7	0.98	0.57–1.69	0.943	97	67.8	46	32.2	0.93	0.58 –1.49	0.763
Pathogen														
* Escherichia coli*	23	24.2	72	75.8	1.00	–	–	93	74.4	32	25.6	1.00	–	–
* Klebsiella pneumoniae*	13	26.5	36	73.5	1.10	0.51–2.35	0.814	48	70.6	20	29.4	1.15	0.61–2.16	0.667
* Pseudomonas aeruginosa*	14	43.8	18	56.2	1.81	0.83–3.93	0.135	23	47.9	25	52.1	2.03	1.09–3.78	**0.025**
* Acinetobacter* spp.	19	65.5	10	34.5	2.71	1.30–5.65	**0.008**	20	57.1	15	42.9	1.67	0.82–3.44	0.160
Origin of infection														
Community	13	19.7	53	80.3	1.00	–	–	61	77.2	18	22.8	1.00	–	–
Hospital	51	44.4	64	55.6	2.25	1.14–4.44	**0.019**	99	60.0	66	40.0	1.76	0.98–3.15	0.06
Neonatal	5	20.8	19	79.2	1.06	0.34–3.28	0.923	24	75.0	8	25.0	1.10	0.43–2.78	0.845
3GC resistance														
No	9	12.5	63	87.5	1.00	–	–	64	66.0	33	34.0	1.00	–	–
Yes	60	45.1	73	54.9	3.61	1.69–7.69	**0.001**	120	67.0	59	33.0	0.97	0.59–1.59	0.900
Carbapenem resistance														
No	36	21.6	131	78.4	1.00	–	–	158	71.2	64	28.8	1.00	–	–
Yes	33	86.8	5	13.2	4.03	2.23–7.26	**< 0.001**	26	48.2	28	51.8	1.80	1.05–3.07	**0.031**
DTR bacteria														
No	47	25.7	136	74.3	1.00	–	–	169	69.3	75	30.7	1.00	–	–
Yes	22	100.0	0	0.00	3.89	1.99–7.63	**< 0.001**	15	46.9	17	53.1	1.73	0.91–3.29	0.095
Inactive empiric treatment														
No	–	–	–	–	–	–	–	95	69.8	41	30.2	1.00	–	–
Yes	–	–	–	–	–	–	–	41	59.4	28	40.6	1.35	0.77–2.36	0.299

3GC = third-generation cephalosporins; DTR = difficult-to-treat resistance; GNB = gram-negative bacteria. A generalized linear model considering the negative binomial discrete distribution and the logarithmic function was used to estimate crude RRs with 95% CIs. *P* < 0.05 was used to determine statistical significance; all values below this cut-off are presented in bold.

### Case fatality.

The overall in-hospital case fatality ratio was 33.3% (92/276), with a median time to death of 3.5 days (IQR: 1–15.5 days) after blood culture sampling and with 66.3% of the deaths occurring within the first 7 days after blood culture sampling. For patients who survived, the median length of hospital stay after blood culture sampling was 14 days (IQR: 9–25 days) (Table 2).

**Figure 3.**
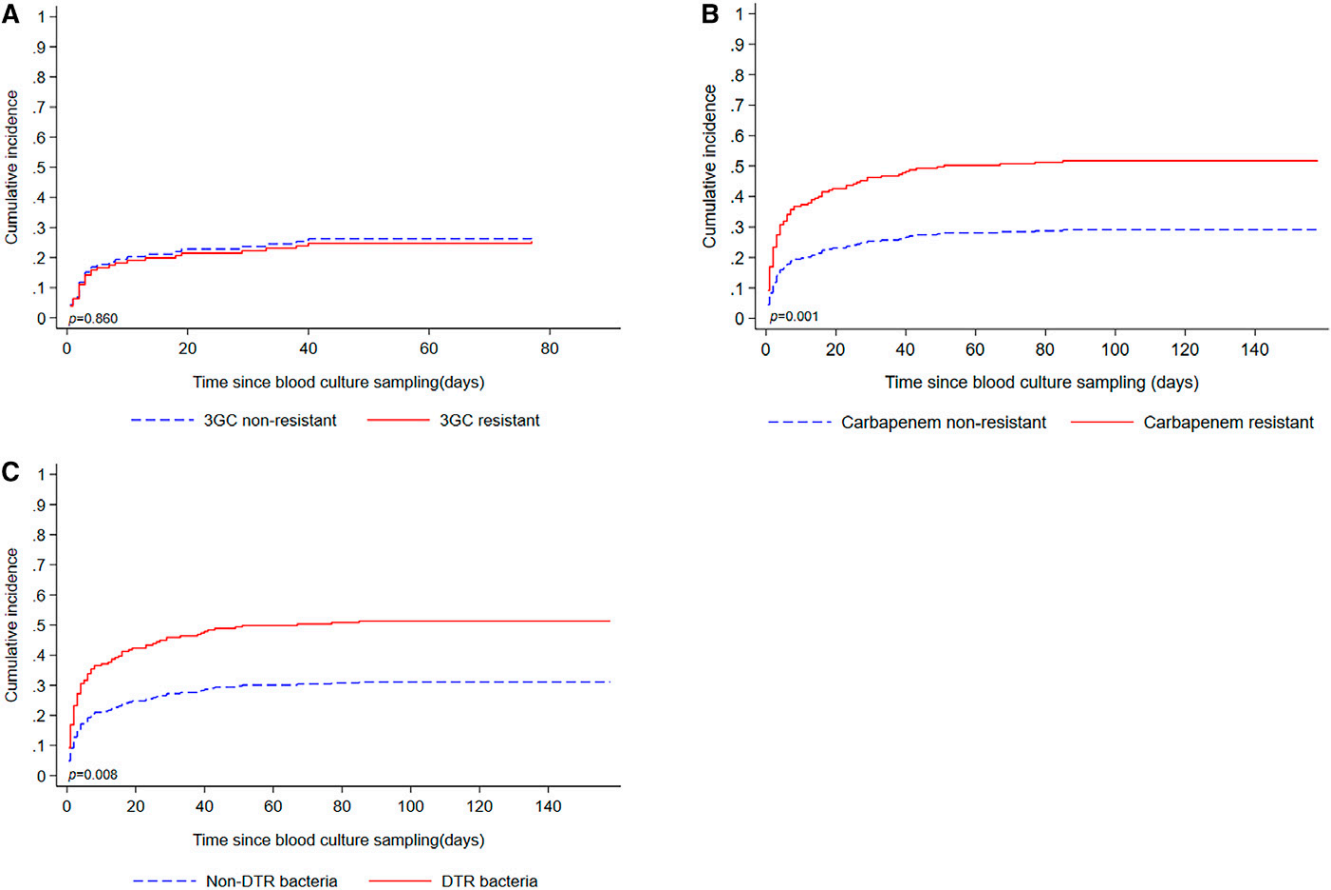
Comparison of the cumulative incidence of in-hospital fatalities after blood culture collection (**A**) between patients with 3GC-resistant and those with nonresistant *Escherichia coli* bloodstream infections, (**B**) between carbapenem-resistant and nonresistant GNB bloodstream infections, and (**C**) between DTR and non-DTR GNB bloodstream infections. 3GC = third-generation cephalosporin; DTR = difficult-to-treat resistance; GNB = gram-negative bacteria.

The risk of death was higher for patients with *P. aeruginosa* bloodstream infection (RR: 2.03, 95%CI: 1.09–3.78; *P* = 0.025) and with carbapenem resistance (RR: 1.79, 95% CI: 1.05–3.06; *P* = 0.033). No higher risk of death was observed among patients infected with GNB resistant to 3GCs (RR: 0.97, 95% CI: 0.6–1.59; *P* = 0.917) or DTR GNB (RR: 1.72, 95% CI: 0.91–3.27; *P* = 0.098) (Table 4). In contrast, an excess in the cumulative incidence of in-hospital fatalities was found for patients with DTR infections (*P* = 0.008) or carbapenem-resistant infections (*P* = 0.001), but not with 3GC-resistant infections (*P* = 0.860) (Figure 3).

## DISCUSSION

This multicenter study provides a comprehensive microbiological, clinical, and epidemiological characterization of bloodstream infections caused by *E. coli*, *K. pneumoniae*, *P. aeruginosa*, and *Acinetobacter* spp. in hospitalized patients across different regions of Peru. Our results show that more than 80% of these infections are antibiotic-resistant infections and 69% are MDR, with a high frequency of inactive empiric treatment and high in-hospital case fatality rate. These findings highlight the urgent need for interventions to improve GNB bloodstream infection management and to control antimicrobial resistance in Peru.

Until recently, national antimicrobial resistance surveillance in Peru was limited to laboratory-based surveillance, as part of the Latin American Antimicrobial Resistance Surveillance Network (ReLAVRA, for its Spanish initials) supported by the Pan American Health Organization. Because this surveillance relies on on-demand isolate referral from hospitals and it includes isolates from any source, it is likely enriched for highly resistant isolates. As an example, in a 2019 ReLAVRA report, carbapenem resistance was present in 41.5%, 77.4%, and 97.8% of *K. pneumoniae*, *P. aeruginosa*, and *Acinetobacter* spp. isolates surveyed in Peru,^21^ whereas our study found carbapenem resistance in 11.0%, 37.0%, and 60.8% of isolates of the same species, respectively.

To foster standardized national surveillance and data sharing in support of global monitoring of antimicrobial resistance, the WHO launched in 2015 the GLASS system.^11^ In 2019, Peru was enrolled in GLASS; however, because of the SARS-CoV-2 pandemic, full implementation was delayed.^2^ By 2022, Peru reported to GLASS for the first-time data on the prevalence of 3GC resistance among *E. coli* bloodstream infections (one of the two antimicrobial resistance indicators of Sustainable Development Goal 3^22^), finding a prevalence of 71.7% among 53 surveyed blood isolates.^23^ In our study, we surveyed a larger set of *E. coli* blood isolates (*n =* 199) and found a similar high prevalence of 3GC resistance (68.3%). This high prevalence is far above the median prevalence of LMICs reported by GLASS (68.3% versus 58.3%) and positions Peru close to the 75th percentile of worst performing LMICs on this antimicrobial resistance indicator.^2^

Likewise, a high frequency of resistance to ACCESS (aminoglycosides and trimethoprim-sulfamethoxazole) and WATCH (ciprofloxacin and 3GC) antibiotics was observed for all four GNB, in many cases higher than the median global ratios reported in GLASS 2021^2^ (Supplemental Table 6).

The high resistance frequencies observed in this study represent an important challenge to select active antibiotics for the empiric treatment of patients with suspected GNB bloodstream infections in Peru. We found that more than one-third of the empiric treatments administered were inactive based on the in vitro antibiotic susceptibility of the recovered isolate. In comparison, other studies conducted in America and Asia have found significantly lower frequencies of inactive empiric treatment of GNB bloodstream infections, ranging from 5.6% to 28.3%.^24^^–^^26^ Although inactive empiric treatment was more frequent for bloodstream infections of hospital origin, one out of five patients with a GNB bloodstream infection of community origin also did not receive active empiric treatment. We also found that infections with resistance to 3GCs or to carbapenems had the highest risk of receiving inactive empiric treatment (45.1% and 86.8%, respectively). Similar ratios of inactive treatment have been reported among ESBL-Enterobacterales bloodstream infections in Colombia and among carbapenem-resistant Enterobacterales bloodstream infections in Argentina (58% and 83.3%, respectively),^27^^,^^28^ but not in an extensive national study in the United States, where inappropriate empiric treatment was found in 26% and 44.7% of ESBL- and carbapenem-resistant Enterobacterales, respectively.^25^ These differences could be due to a number of factors, including limited access to newer antibiotics or dated or nonexistent local treatment guidelines; however, further studies are needed to better understand the underpinning of these differences.

Considering that early administration of an active antibiotic for treatment of bloodstream infections strongly correlates with survival,^29^^,^^30^ the implications of these findings are serious and warrant immediate interventions to improve patients’ outcomes. This study provides a glimpse of the elevated burden of disease of GNB bloodstream infections in Peru. One out of three patients hospitalized with a GNB bloodstream infection died during their hospitalization (crude mortality was 33.3%). This in-hospital mortality rate is higher than the 15.0% to 26.3% mortality rate reported in some other GNB bloodstream infection studies.^8^^,^^31^^,^^32^ The higher prevalence of carbapenem resistance, MDR, and DTR, as well as the high frequency of inactive empiric treatment found in this study could explain this increased mortality, because all of these factors were previously found to be associated with increased mortality.^31^^–^^33^ However, establishing the attribution of these factors to mortality goes beyond the scope of this study and will require future studies specifically designed and powered to assess this.

One of the main limitations of this study is that the number of blood culture isolates recovered was significantly lower than expected. This early finding prompted a parallel quality assessment of blood culture utilization and processing in these hospitals, finding important shortcomings in several quality indicators, as recently published.^34^ One of these shortcomings is the high ratio of blood culture contamination (measured using the ratio of coagulase-negative staphylococci growth as a proxy), which was found to be 7.7% when the recommended target is 3% or less.^35^ Another important shortcoming found in the prior study was that there is a significant underutilization of blood cultures in Peruvian hospitals.^34^ This could lead to important selection bias, as it is possible that blood cultures were requested more frequently in patients who were failing treatment or who had a more severe presentation. This in turn could result in an overestimation of antimicrobial resistance prevalence and mortality. Importantly, this is not a specific problem of the current study, but a problem embedded in Peru’s hospital system and possibly in other LMICs.^36^^–^^38^ Therefore, the interpretations of these results and any results obtained with the current national surveillance system should be done with this in mind, especially when used for comparisons with other countries with better blood culture utilization and quality.

The resulting small number of blood culture isolates, especially in hospitals from regions outside Lima and in the pediatric population, significantly limited our capacity to obtain reliable antimicrobial resistance ratios for certain populations (i.e., neonatal, pediatric) and for certain regions (those with less than 30 isolates per region) and to conduct subgroup comparisons or multivariate analysis.

Lack of electronic medical records at public hospitals in Peru limited our capacity to collect clinical data, requiring manual retrieval and review of paper charts, which resulted in obtaining clinical data in only 60% of the source patients. In addition, we could not include as an aim of our study an estimation of the incidence of bloodstream infections because obtaining aggregated data on the number of hospital admissions or the number of episodes of sepsis during the study period at each participating hospital was unattainable owing to a lack of electronic medical systems at the participating hospitals.

Although these are limitations of the current study, they also reflect the reality of bloodstream infection diagnosis and management in Peru, and likely other LMICs. Lack of electronic medical records hinders clinical and epidemiological research, whereas underutilization of blood cultures and shortcomings in the quality of clinical microbiological laboratories result in suboptimal patient management and hinder local and national antimicrobial resistance surveillance.

On the other hand, this study has several strengths. First, the systematic collection of consecutive blood isolates at 15 of the largest tertiary hospitals located in 12 of 24 geographic regions provides data on the diagnosed GNB bloodstream infections in Peru with relevant geographic representativity. Second, centralizing antimicrobial susceptibility testing ensured harmonized quality-assured data and allowed for testing of additional antibiotics. Third, by performing a systematic collection of linked epidemiological data, the present study provides valuable data for estimation of antimicrobial resistance distribution and burden in different population subgroups.

The present data will support antibiotic stewardship efforts in Peru, which have been started up and were recently leveraged by the approval of a national norm for implementation of hospital Antibiotic Stewardship programs.^39^ Moreover, because this study was performed between 2017 and 2019, its results may serve as a pre–COVID-19 baseline to assess the effect of the pandemic on antimicrobial resistance in our country and, in the longer term, to monitor the effect of the implementation of the Antibiotic Stewardship national norm. Future studies should expand antimicrobial resistance data on specific populations, such as the neonatal and pediatric populations. Finally, this study reinforces the need to regain momentum with the implementation of a continuous, representative, and high-quality antimicrobial resistance surveillance system in Peru.

In conclusion, a high prevalence of antimicrobial resistance was observed among the four most common GNB causing bloodstream infections in Peru, even when these infections originated in the community. Importantly, the prevalence of 3GC resistance found among *E. coli* blood isolates, a sustainable development goal indicator, was at the higher end of ratios for LMICs in 2020. The high prevalence of antimicrobial resistance, high prevalence of inactive empiric treatment, and high mortality rate found in this study highlight the need for implementation of stronger diagnostic and antibiotic stewardship strategies and a high-quality national antimicrobial resistance surveillance system.

## Supplemental Materials


Supplemental materials

## References

[1] Collaborators AR, 2022. Global burden of bacterial antimicrobial resistance in 2019: a systematic analysis. Lancet 399: 629–655. 35065702 10.1016/S0140-6736(21)02724-0 PMC8841637

[2] World Health Organization , 2021. Global Antimicrobial Resistance and Use Surveillance System (GLASS) Report 2021. Geneva, Switzerland: WHO.

[3] Jasovsky D Littmann J Zorzet A Cars O, 2016. Antimicrobial resistance-a threat to the world’s sustainable development. Ups J Med Sci 121: 159–164. 27416324 10.1080/03009734.2016.1195900 PMC4967260

[4] O’Neill J, 2016. Tackling Drug-Resistant Infections Globally: Final Report and Recommendations. London, United Kingdom: Government of the United Kingdom.

[5] Tacconelli E , 2018. Discovery, research, and development of new antibiotics: the WHO priority list of antibiotic-resistant bacteria and tuberculosis. Lancet Infect Dis 18: 318–327. 29276051 10.1016/S1473-3099(17)30753-3

[6] World Health Organization , 2017. Prioritization of Pathogens to Guide Discovery, Research and Development of New Antibiotics for Drug-Resistant Bacterial Infections, Including Tuberculosis. Geneva, Switzerland: WHO.

[7] Effah CY , 2021. Evaluation of the therapeutic outcomes of antibiotic regimen against carbapenemase-producing *Klebsiella pneumoniae*: a systematic review and meta-analysis. Front Pharmacol 12: 597907. 34803661 10.3389/fphar.2021.597907 PMC8599800

[8] Kadri SS , 2018. Difficult-to-treat resistance in gram-negative bacteremia at 173 US hospitals: retrospective cohort analysis of prevalence, predictors, and outcome of resistance to all first-line agents. Clin Infect Dis 67: 1803–1814. 30052813 10.1093/cid/ciy378 PMC6260171

[9] Chow JW Yu VL, 1999. Combination antibiotic therapy versus monotherapy for gram-negative bacteraemia: a commentary. Int J Antimicrob Agents 11: 7–12. 10075272 10.1016/s0924-8579(98)00060-0

[10] Singer M , 2016. The Third International Consensus Definitions for Sepsis and Septic Shock (Sepsis-3). JAMA 315: 801–810. 26903338 10.1001/jama.2016.0287 PMC4968574

[11] World Health Organization , 2015. Global Antimicrobial Resistance Surveillance System: Manual for Early Implementation. Geneva, Switzerland: WHO.

[12] World Health Organization , 2021. *WHO Access, Watch, Reserve (AWaRe) Classification of Antibiotics for Evaluation and Monitoring of Use, 2021*. Available at: https://apps.who.int/iris/handle/10665/345555. Accessed August 10, 2022.

[13] Koneman EW Janda WM Schreckenberger PC Winn WC, 2004. Diagnóstico Microbiológico Texto y Atlas en color, 5th edition. Buenos Aires, Argentina: Editorial Médica Panamericana S.A.

[14] Turton JF Woodford N Glover J Yarde S Kaufmann ME Pitt TL, 2006. Identification of *Acinetobacter baumannii* by detection of the bla_OXA-51-like_ carbapenemase gene intrinsic to this species. J Clin Microbiol 44: 2974–2976. 16891520 10.1128/JCM.01021-06 PMC1594603

[15] Clinical and Laboratory Standards Institute , 2017. Performance Standards for Antimicrobial Susceptibility Testing, 27th edition. CLSI Supplement M100. Wayne, PA: CLSI.

[16] Clinical and Laboratory Standards Institute , 2021. Performance Standards for Antimicrobial Susceptibility Testing, 31st edition. CLSI Supplement M100. Wayne, PA: CLSI. 10.1128/JCM.00213-21 PMC8601225 34550809

[17] The European Committee on Antimicrobial Susceptibility Testing , 2021. *Breakpoint Tables for Interpretation of MICs and Zone Diameters, Version 11.0, 2021*. Available at: https://www.eucast.org/fileadmin/src/media/PDFs/EUCAST_files/Breakpoint_tables/v_11.0_Breakpoint_Tables.pdf. Accessed February 21, 2022.

[18] Wyeth Pharmaceuticals Inc Tygacil® (tigecycline) for Injection. Philadelphia, PA: Wyeth Pharmaceuticals Inc. Available at: https://www.accessdata.fda.gov/drugsatfda_docs/label/2010/021821s021lbl.pdf. Accessed February 21, 2022.

[19] Servicio Antimicrobianos, Laboratorio Nacional de Referencia en Antimicrobianos, INEI-ANLIS, “Dr. Carlos G. Malbrán” , 2017. *Método de Screening “COLISTIN AGAR-SPOT” Version 2*. Available at: http://antimicrobianos.com.ar/ATB/wp-content/uploads/2017/09/Protocolo-Agar-spot-COL-2017-version2-Agosto2017.pdf. Accessed March 16, 2018.

[20] Magiorakos AP , 2012. Multidrug-resistant, extensively drug-resistant and pandrug-resistant bacteria: an international expert proposal for interim standard definitions for acquired resistance. Clin Microbiol Infect 18: 268–281. 21793988 10.1111/j.1469-0691.2011.03570.x

[21] Pan American Health Organization, 2023. *Health Information Platform for the Americas* Available at: https://www3.paho.org/data/index.php/en/mnu-topics/antimicrobial-resistance/571-amr-vig-en.html. Accessed August 10, 2022.

[22] United Nations , 2021. Sustainable Development Goals Indicator Metadata. Available at: https://unstats.un.org/sdgs/metadata/files/Metadata-03-0D-02.pdf. Accessed June 4, 2023.

[23] World Health Organization , 2022. Global Antimicrobial Resistance and Use Surveillance System (GLASS): 2022 Report. Available at: https://worldhealthorg.shinyapps.io/glass-dashboard/_w_069e9761/_w_64f43738/#!/cta-profiles. Accessed June 4, 2023.

[24] Kadri SS , 2021. Inappropriate empirical antibiotic therapy for bloodstream infections based on discordant in-vitro susceptibilities: a retrospective cohort analysis of prevalence, predictors, and mortality risk in US hospitals. Lancet Infect Dis 21: 241–251. 32916100 10.1016/S1473-3099(20)30477-1 PMC7855478

[25] Ohnuma T Chihara S Costin B Treggiari MM Bartz RR Raghunathan K Krishnamoorthy V, 2023. Association of appropriate empirical antimicrobial therapy with in-hospital mortality in patients with bloodstream infections in the US. JAMA Netw Open 6: e2249353. 36598788 10.1001/jamanetworkopen.2022.49353 PMC9857618

[26] Xu S Song Z Han F Zhang C, 2023. Effect of appropriate empirical antimicrobial therapy on mortality of patients with gram-negative bloodstream infections: a retrospective cohort study. BMC Infect Dis 23: 344. 37221465 10.1186/s12879-023-08329-2 PMC10204198

[27] Arias Ramos D Alzate JA Moreno Gomez GA Hoyos Pulgarin JA Olaya Gomez JC Cortes Bonilla I Vargas Mosquera C, 2023. Empirical treatment and mortality in bacteremia due to extended spectrum beta-lactamase producing Enterobacterales (ESBL-E), a retrospective cross-sectional study in a tertiary referral hospital from Colombia. Ann Clin Microbiol Antimicrob 22: 13. 36797734 10.1186/s12941-023-00566-2 PMC9933341

[28] Lipari FG Hernández D Vilaró M Caeiro JP Saka HA, 2020. Caracterización clínica, epidemiológica y microbiológica de bacteriemias producidas por enterobacterias resistentes a carbapenems en un hospital universitario de Córdoba, Argentina. Rev Chilena Infectol 37: 362–370. 33399656 10.4067/S0716-10182020000400362

[29] Rhodes A , 2017. Surviving sepsis campaign: international guidelines for management of sepsis and septic shock: 2016. Intensive Care Med 43: 304–377. 28101605 10.1007/s00134-017-4683-6

[30] Cain SE Kohn J Bookstaver PB Albrecht H Al-Hasan MN, 2015. Stratification of the impact of inappropriate empirical antimicrobial therapy for gram-negative bloodstream infections by predicted prognosis. Antimicrob Agents Chemother 59: 245–250. 25348527 10.1128/AAC.03935-14 PMC4291357

[31] Huh K , 2020. Impact of difficult-to-treat resistance in gram-negative bacteremia on mortality: retrospective analysis of nationwide surveillance data. Clin Infect Dis 71: e487–e496. 31994704 10.1093/cid/ciaa084

[32] Falcone M , 2023. Mortality attributable to bloodstream infections caused by different carbapenem-resistant gram-negative bacilli: results from a nationwide study in Italy (ALARICO Network). Clin Infect Dis 76: 2059–2069. 36801828 10.1093/cid/ciad100

[33] Stewardson AJ , 2019. Effect of carbapenem resistance on outcomes of bloodstream infection caused by Enterobacteriaceae in low-income and middle-income countries (PANORAMA): a multinational prospective cohort study. Lancet Infect Dis 19: 601–610. 31047852 10.1016/S1473-3099(18)30792-8

[34] Krapp F , 2021. Underutilization and quality gaps in blood culture processing in public hospitals of Peru. Am J Trop Med Hyg 106: 432–440. 34872054 10.4269/ajtmh.21-0770 PMC8832895

[35] Clinical and Laboratory Standards Institute , 2007. Principles and Procedures for Blood Cultures; Approved Guideline. CLSI document M47-A. Wayne, PA: CLSI.

[36] Ombelet S Ronat J-B Walsh T Yansouni CP Cox J Vlieghe E Martiny D Semret M Vandenberg O Jacobs J; Bacteriology in Low Resource Settings Working Group , 2018. Clinical bacteriology in low-resource settings: today’s solutions. Lancet Infect Dis 18: e248–e258. 29519767 10.1016/S1473-3099(18)30093-8

[37] Lim C , 2021. Surveillance strategies using routine microbiology for antimicrobial resistance in low- and middle-income countries. Clin Microbiol Infect 27: 1391–1399. 34111583 10.1016/j.cmi.2021.05.037 PMC7613529

[38] Gandra S Alvarez-Uria G Turner P Joshi J Limmathurotsakul D van Doorn HR, 2020. Antimicrobial resistance surveillance in low- and middle-income countries: progress and challenges in eight South Asian and Southeast Asian Countries. Clin Microbiol Rev 33: e00048-19. 32522747 10.1128/CMR.00048-19 PMC7289787

[39] MINSA/DIGEMID , 2022. Norma Técnica de Salud para la Implementación del Programa de Optimización del Uso de Antimicrobianos a Nivel Hospitalario. NTS N°184-MINSA/DIGEMID-2022. Lima, Peru: MINSA. Available at: https://cdn.www.gob.pe/uploads/document/file/2878122/NTS%20Nº%20184-MINSA/DIGEMID-2022.pdf?v=1646484067. Accessed June 4, 2023.

