# Supplementary material for: Prevalence of Antimicrobial Resistance in Gram-Negative Bacteria Bloodstream Infections in Peru and Associated Outcomes: VIRAPERU Study
Source: Am J Trop Med Hyg. 2023 Sep 18;109(5):1095–106. doi: 10.4269/ajtmh.22-0556 (PMC10622474; doi:10.4269/ajtmh.22-0556)
Supplement: Supplementary file 1 [file tpmd220556.SD1.pdf]

Table S1. Standardized panels of antibiotic susceptibility testing performed in the reference laboratory to surveilled isolates according to the species. The method used for antibiotic susceptibility testing and the breakpoints used for interpretation of results are also listed.

Escherichia coli	***Klebsiella pneumoniae***	***Pseudomonas aeruginosa***	***Acinetobacter spp.***	***Method***	***Breakpoints***
Gentamicin	Gentamicin	Gentamicin	Gentamicin	Disk diffusion	CLSI 2021
Amikacin	Amikacin	Amikacin	Amikacin	Disk diffusion	CLSI 2021
Piperacillin-tazobactam	Piperacillin-tazobactam	Piperacillin-tazobactam	Ertapenem	Disk diffusion	CLSI 2021
Ertapenem	Ertapenem	Ertapenem	Imipenem	Disk diffusion	CLSI 2021
Imipenem	Imipenem	Imipenem	Meropenem	Disk diffusion	CLSI 2021
Meropenem	Meropenem	Meropenem		Disk diffusion	CLSI 2021
Cefazolin	Cefazolin			Disk diffusion	CLSI 2021
Ceftriaxone	Ceftriaxone			Disk diffusion	CLSI 2021
Ceftazidime	Ceftazidime	Ceftazidime	Ceftazidime	Disk diffusion	CLSI 2021
Cefepime	Cefepime	Cefepime		Disk diffusion	CLSI 2021
Ciprofloxacin	Ciprofloxacin	Ciprofloxacin	Ciprofloxacin	Disk diffusion	CLSI 2021
TMP/SMX	TMP/SMX		TMP/SMX	Disk diffusion	CLSI 2021
Tigecycline	Tigecycline		Tigecycline	Disk diffusion	FDA
Aztreonam	Aztreonam	Aztreonam		Disk diffusion	CLSI 2021
Fosfomycin	Fosfomycin	Fosfomycin [a]		Disk diffusion	CLSI 2021, EUCAST 2021 [a]
			Ampicillin-sulbactam	Disk diffusion	CLSI 2021
			Minocycline	Disk diffusion	CLSI 2021
	Colistin [b]	Colistin [b]	Colistin [b]	Disk ellution	CLSI 2021
	Colistin [b]	Colistin [b]	Colistin [b]	Agar spot	CLSI 2021
ESBL production [c]	ESBL production [c]	ESBL production [c]	ESBL production [c]	Double disk	CLSI 2021

Abbreviations: TMP/SMX, Trimethoprim-sulfamethoxazole; CLSI, Clinical and Laboratory Standards Institute; FDA, U.S. Food and Drug Administration; EUCAST, The European Committee on Antimicrobial Susceptibility Testing; ESBL, extended spectrum beta-lactamase.

[a] For fosfomycin, 2021 CLSI breakpoints were used for *E. coli* and extrapolated to *K. pneumoniae*. For P. aeruginosa, the 2021 EUCAST epidemiologic cutoff value of 12mm was used.

[b] Colistin susceptibility testing was only performed in carbapenem-resistant isolates

[c] ESBL production was tested only in isolates with resistance to third generation cephalosporins

Table S2. Number of antibiotic susceptibility tests conducted per antibiotic and per species.

		Number of susceptibility tests performed				
**Antibiotic classes**	**Antibiotics**	**Total**	***Escherichia coli***	***Klebsiella pneumoniae***	***Pseudomonas aeruginosa***	***Acinetobacter spp.***
Aminoglycosides	Gentamicin	449	199	118	81	51
	Amikacin	449	199	118	81	51
Antipseudomonals + beta-lactamase inhibitor	Piperacillin-tazobactam	398	199	118	81	-
Carbapenems	Ertapenem	449	199	118	81	51
	Imipenem	449	199	118	81	51
	Meropenem	448	198	118	81	51
Non-extended spectrum cephalosporins	Cefazolin	317	199	118	-	-
Extended spectrum cephalosporins	Ceftriaxone	317	199	118	-	-
	Ceftazidime	449	199	118	81	51
	Cefepime	398	199	118	81	-
Fluoroquinolones	Ciprofloxacin	449	199	118	81	51
Folate pathway inhibitor	Trimethoprim-sulfamethoxazole	368	199	118	-	51
Glycylcyclines	Tigecycline	368	199	118	-	51
Monobactams	Aztreonam	398	199	118	81	-
Penicillins + beta-lactamase inhibitor	Ampicillin-sulbactam	51	-	-	-	51
Phosphonic acids	Fosfomycin	397	199	117	81	-
Tetracyclines	Minocycline	51	-	-	-	51
Polymyxins	Colistin	74	-	13	30	31
**Total**		**6279**	**2984**	**1782**	**921**	**592**

Table S3. Comparison of the frequencies of resistance to different antibiotic classes between surveilled blood isolates of key Gram-negative bacteria recovered in hospitals in Lima and Callao macroregion with isolates recovered in other macroregions of Peru. *Acinetobacter* spp. isolates had higher frequency of resistance to different antibiotics in Lima compared with the rest of the regions. *Klebsiella pneumoniae* isolates had higher frequency of carbapenem resistance compared with the rest of the regions, with only 2 carbapenem resistant isolates found outside Lima, both in the North macroregion. The numbers presented for each macroregion are absolute frequencies. The relative frequencies for each macroregion were not estimated due to the small number of isolates available for most of the macroregions.

Resistance profile[a]	Lima and Callao (N=280)		All other regions[b] (N=169)		*p*	Macroregion North (N=86)	South (N=42)	Center (N=6)	Jungle (N=35)
	n	(%)	n	(%)		n	n	n	n
Escherichia coli									
Total	128		71			34	22	2	13
3GC	86	(67.2)	50	(70.4)	0.751	20	17	1	12
Carbapenems	0	(0.0)	0	(0.0)	–	0	0	0	0
Fluoroquinolones	96	(75.0)	54	(76.1)	1.000	21	20	1	12
Aminoglycosides	50	(39.1)	30	(42.3)	0.763	17	5	2	6
Difficult-to-treat resistance	0	(0.0)	0	(0.0)	–				
Klebsiella pneumoniae									
Total	75		43			23	11	1	8
3GC	51	(68.0)	30	(69.8)	1.000	17	8	1	4
Carbapenems	11	(14.7)	2	(4.7)	0.129	2	0	0	0
Fluoroquinolones	52	(69.3)	28	(65.1)	0.685	14	8	1	5
Aminoglycosides	40	(53.3)	19	(44.2)	0.444	11	5	1	2
Difficult-to-treat resistance	10	(13.2)	1	(2.3)	0.054	1	0	0	0
Pseudomonas aeruginosa									
Total	45		36			24	4	2	6
3GC	10	(22.2)	6	(16.7)	0.586	3	2	0	1
Carbapenems	16	(35.6)	14	(38.9)	0.819	8	2	2	2
Fluoroquinolones	12	(26.7)	10	(27.8)	1.000	4	2	2	2

Aminoglycosides	11	(24.4)	11	(30.6)	0.619	6	2	2	1
Difficult-to-treat resistance	4	(8.9)	6	(16.7)	0.327	3	2	0	1
Acinetobacter spp.									
Total	32		19			5	5	1	8
3GC	24	(75.0)	9	(47.4)	0.070	2	4	1	2
Carbapenems	22	(68.8)	9	(47.4)	0.151	2	4	1	2
Fluoroquinolones	25	(78.1)	8	(42.1)	**0.015**	2	4	1	1
Aminoglycosides	24	(75.0)	7	(36.8)	**0.009**	1	4	1	1
Difficult-to-treat resistance	19	(59.4)	6	(31.6)	0.083	2	3	1	0

Abbreviations: 3GC, third generation cephalosporins

[a] Resistance to third generation cephalosporins was defined as resistance to ceftriaxone or ceftazidime for *Escherichia coli* and *Klebsiella pneumoniae*; and as resistance to ceftazidime for *Pseudomonas aeruginosa* and *Acinetobacter* spp. Resistance to fluoroquinolones was defined as resistance to ciprofloxacin. Resistance to aminoglycosides was defined as resistance to amikacin or gentamicin.

[b] Includes the North, South, Central and Jungle macro-regions

Fisher's exact test was used for the comparison between Lima and other regions. A p-value of <0.05 was used to determine statistical significance.

Table S4. Proportion of patients with clinical data available by age, sex and pathogen. No significant difference in the proportions of patients with missing clinical data was observed between the analyzed categories.

**Characteristics**	**Included to clinical analysis (N = 276)**		**Missing clinical data (N = 173)**		***p-value***
	**n**	**(%)**	**n**	**(%)**	
Age					
Neonatal	32	(62.8)	19	(37.2)	0.626*
Pediatric	20	(71.4)	8	(28.6)	
Adult	224	(61.7)	139	(38.3)	
Unknown	0	(0.0)	7	(100.0)	
Sex					
Female	133	(63.9)	75	(36.1)	0.843*
Male	143	(63.0)	84	(37.0)	
Unknown	0	(0.0)	14	(100.0)	
Pathogen					0.546
E. coli	125	(62.8)	74	(37.2)	
K. pneumoniae	68	(57.6)	50	(42.4)	
P. aeruginosa	48	(59.3)	33	(40.7)	
Acinetobacter spp	35	(68.6)	16	(31.4)	

Fisher's exact test was used for the comparison. A p-value of <0.05 was used to determine statistical significance.
*The comparison excluding the 'unknown' category.

Table S5. Characteristics of the eight possible clusters identified, including the hospital and ward where the cluster was identified; the species of GNB involved in the cluster, as well as the resistance profile of the clustering isolates; and the number of isolates. Possible clustering was evaluated by identifying isolates of the same species that were recovered in the same ward within a 14-day span and had similar resistance profiles. The exact number of days elapsed between the recovery of each isolate of the cluster is provided in the table.

**Cluster code**	**Hospital code**	**Hospital ward were possible cluster was identified**	**Clustering microorganism**	**Nº of isolates clustering**	**Nº of days between recovery of each isolate**	**Resistance profile**				
						**3GC**	**CARB**	**FQ**	**AG**	**DTR**
Cluster 1	V14	Neonatal	*Acinetobacter baumannii*	2	5	S	S	S	S	-
Cluster 2	V01	Infectious diseases	*Pseudomonas aeruginosa*	2	4	S	S	S	S	-
Cluster 3	V12	Medicine	*Pseudomonas aeruginosa*	2	0	S	S	S	S	-
Cluster 4	V13	Medicine	*Pseudomonas aeruginosa*	2	4	S	S	S	S	-
Cluster 5	V01	Medicine	*Klebsiella pneumoniae*	2	5	R	R	R	R	+
Cluster 6	V14	Nephrology	*Klebsiella pneumoniae*	2	12	R	S	R	R	-
Cluster 7	V01	Medicine	*Escherichia coli*	2	2	R	S	R	R	-
Cluster 8	V01	Infectious diseases	*Escherichia coli*	2	1	R	S	R	R	-

Abbreviations: GNB, Gram-negative bacteria; 3GC, third generation cephalosporins; CARB, carbapenems; FQ, fluoroquinolones; AG, aminoglycosides; DTR, difficult-to-treat resistance; S, susceptible; R, resistant

Table S6. Comparison of antimicrobial resistance rates found in this study with other national and international surveillance reports and studies.

Resistance profile[a]	Rates of resistance (%)					
	At Peruvian national level			Compared with international reports		
	**Current study (2017-2019)**	**ReLAVRA [19] (2019)**	**AMR GBD Study [1] (2019)**	**ReLAVRA [19]-South America countries (2019)**	**GLASS [2] (2021)**	**Al-Hasan M [20] (2021)**
Source of isolates	**Blood**	All	All	All	Blood	Blood
Escherichia coli						
3GC	**68.3**		40 - <50		58 (40-70)†	
Carbapenems	**0**				1 (0-5)	
Fluoroquinolones	**75.4**		50 - <60		41 (24-50)	
Aminoglycosides	**40.2**					
Difficult-to-treat resistance	**0**					0 - 0.07
Klebsiella pneumoniae						
3GC	**68.6**	77.7	60 - <70	34.1 - 77.7	55 (27-78)	
Carbapenems	**11**	41.5	≤5	0.0 - 41.5	17 (3-33)	
Fluoroquinolones	**67.8**	57.6		3.5 - 65.9	41 (18-58)	
Aminoglycosides	**50**	48.2		6.9 - 69.4		
Difficult-to-treat resistance	**9.3**					1.4 - 2.8
Pseudomonas aeruginosa						
3GC	**19.8**	70.7		11.4 - 70.7		
Carbapenems	**37**	77.4		21.2 - 77.4		
Fluoroquinolones	**27.2**	68.6		23.6 - 68.6		
Aminoglycosides	**27.2**	49.7		8.2 - 49.7		
Difficult-to-treat resistance	**12.4**					2.3 - 9.0
Acinetobacter spp.						
3GC	**64.7**	97.9		11.3 - 97.9		
Carbapenems	**60.8**	97.8	≥80	0.0 - 97.8	65.5 (10-79)	
Fluoroquinolones	**64.7**					
Aminoglycosides	**60.8**	97.1		0.0 - 97.1	50 (13-71)	

Difficult-to-treat resistance	**49**	17.2 - 59.4

Abbreviations: 3GC, third generation cephalosporins; ReLAVRA, Latin American AMR Surveillance Network; AMR GBD; Global burden of bacterial antimicrobial resistance in 2019: a systematic analysis; GLASS, Global Antimicrobial Resistance and Use Surveillance System.
